# Prevalence of genetic mutations in Alpha-1 antitrypsin in patients with difficult-to-treat asthma in Colombia

**DOI:** 10.1186/s12890-025-03840-5

**Published:** 2025-08-08

**Authors:** Abraham Alí-Munive, Jaime Leonardo Chacón, Leidy Prada, Nadia Juliana Proaños, Leslie Vargas, Claudia Diaz-Bossa, Angela Giraldo, John Pedrozo-Pupo

**Affiliations:** 1 Fundación Neumológica Colombiana,; 2 Universidad de La Sabana, Chia, Cundinamarca 111321 Colombia; 3 Instituto Neumológico del Oriente, Bucaramanga, Santander 680001 Colombia; 4 Neumomed IPS, Medellin, Antioquia Colombia; 5 CardioSalud, Pereira, Risaralda Colombia; 6 Respire - Instituto para el cuidado respiratorio, Santa Marta, Magdalena Colombia

**Keywords:** Alpha 1- antitrypsin deficiency, Asthma, Genetic mutation, Genotyping test, Epidemiology, Prevalence

## Abstract

**Background:**

Alpha-1 antitrypsin (AAT) is a medium-sized globular glycoprotein distributed in serum and tissues. In the lungs, it inhibits serine proteases and has anti-inflammatory properties in different types of cells, protecting lung tissue from damage. Mutations in the *SERPINA1* gene that codes for AAT are related to asthma and chronic obstructive pulmonary disease. In Colombia, there are no published data on the prevalence of alpha-1 antitrypsin deficiency (AATD) in adult patients with difficult-to-manage asthma. ​This study aims to determine the prevalence of genetic mutations related to AAT in adult patients with difficult-to-treat asthma in Colombia.

**Methods:**

This prospective, multicenter, cross-sectional study included adult patients with difficult-to-treat asthma in five asthma care centers in Colombia. Informed consent was obtained, and gingival mucosa was sampled for genetic diagnosis of AATD using the A1AT Genotyping Test. Data analysis was performed using the Chi^2^ test, Student’s t-test, and Mann-Whitney test for group comparison.

**Results:**

A total of 449 adult patients with difficult-to-treat asthma were included with a mean age of 56.1 ± 14.9 years; 73.1% (*N* = 328) were women; and 89.1% were using high-dose inhaled corticosteroid / long-acting B2 agonists. Mutations in the AAT gene were found in 12.5% (*N* = 56) of patients. Of these, 85.7% had the M/S genotype, 10.7% the M/Z genotype, 1.8% the M/I genotype, and 1.8% the S/S genotype.

**Conclusions:**

The study identified a prevalence of AAT mutations in 12.5% of adult patients with difficult-to-treat asthma in Colombia made up of four genotypes: M/S, M/Z, M/I and S/S.

## Introduction

Alpha-1 antitrypsin (AAT) is a glycoprotein belonging to the serpin protein superfamily, which acts as a protease inhibitor. In the human body, approximately 40% of AAT is found in serum and 60% in extracellular tissues [1]. AAT is synthesized and secreted mainly in hepatocytes, although it is also produced by monocytes, macrophages, enterocytes, alpha and delta cells of the pancreas, keratinocytes of the cornea, and alveolar epithelial type II cells of the lung [2]. 

At the pulmonary level, the main function of AAT is to inhibit neutrophil serine proteases, especially neutrophil elastase. In addition, AAT has anti-inflammatory properties in different cell types and modulates inflammation caused by both host and microbial factors, thus protecting lung tissue from damage. Mutations in the AAT gene can lead to difficult-to-manage respiratory diseases such as asthma and chronic obstructive pulmonary disease (COPD) [3, 4]. 

In Colombia, the prevalence of asthma is 9%, and 56.6% of these patients do not have adequate control of their symptoms [5]. It is estimated that up to 10.5% of patients with asthma may have mutations in the AAT gene [3, 6]. However, there is no published data on the prevalence of AAT mutations in patients with difficult-to-manage asthma in Colombia. Therefore, the aim of this study was to determine the prevalence of genetic mutations and genotypes present in AAT deficiency (AATD) in adults with difficult-to-treat asthma in five reference centers in Colombia.

## Methods

### Study design and population

This prospective, multicenter, cross-sectional study included adult patients with difficult-to-manage asthma in five comprehensive care centers located in the cities of Santa Marta, Medellín, Pereira, Bucaramanga and Bogotá, Colombia.

Adult patients diagnosed with difficult-to-treat asthma were consecutively recruited in the specialized care clinics of the participating centers between January 1, 2023, and June 30, 2024. The inclusion criteria for the study were: patients over 18 years of age with a previous diagnosis of asthma confirmed by pre-bronchodilator spirometry with a forced expiratory volume in the first second (FEV1) to the forced vital capacity (FVC) ratio indicative of reversible obstruction [7], measured in the previous two years. In addition, patients had to have a diagnosis of difficult-to-treat asthma defined as asthma in steps 3 to 5 of treatment according to the Global Initiative for Asthma (GINA) 2024 guidelines [7]. Eligible patients also had poor symptom control despite an adequate therapeutic strategy adjusted to the level of clinical severity, an absence of symptoms of lower or upper respiratory tract infection for at least four weeks prior to inclusion, and had not used antibiotics or steroids in that period. Exclusion criteria included having a previous AAT genotyping study and the patient’s lack of authorization for the sample to be taken.

### Sample size

The sample size calculation was performed to obtain a prevalence or proportion, with a confidence interval (CI) of 95%, a precision of 2%, and an estimated prevalence of AATD phenotypes in patients with difficult-to-control asthma of 4.9% [6, 8]. A total of 448 patients were initially considered for the study.

### Data collection and management

The clinical histories of the patients who met the selection criteria to participate in the study and who signed informed consent for the collection of samples for genotypic analysis were reviewed. The data of interest were collected from digital clinical histories, including the results from the scales for assessing asthma control [9–12], which are widely recognized tools. The data were entered into the REDCap data capture software, maintaining confidentiality [13]. An oral sample collector was used to collect the gingival mucosa sample. The genetic diagnosis of the AATD was performed using the A1AT Genotyping Test (Progenika Clinical Diagnostic Laboratory, Bizkaia Technology Park, Derio, Spain, a Grifols company) [14].

### Statistical analysis

Qualitative variables were described in absolute and relative frequencies. Quantitative variables were described by measures of central tendency and dispersion and according to their distribution evaluated by the Kolmogorov-Smirnov test for the comparison of groups with and without AATD. The Chi^2^ test was used for qualitative variables and the Student t-test or the Mann-Whitney U test for continuous variables, depending on their distribution. All statistical tests were two-tailed, with a 95% CI and a *P-*value of 0.05 considered statistically significant. The data were analyzed using the statistical software RStudio and Stata 16 [15]. 

### Ethical considerations

This study followed the standards of Good Clinical Practices and the Declaration of Helsinki, (version 2013), additionally it was approved by the ethics committee of the Fundación Neumológica Colombiana.

## Results

**Fig. 1:**
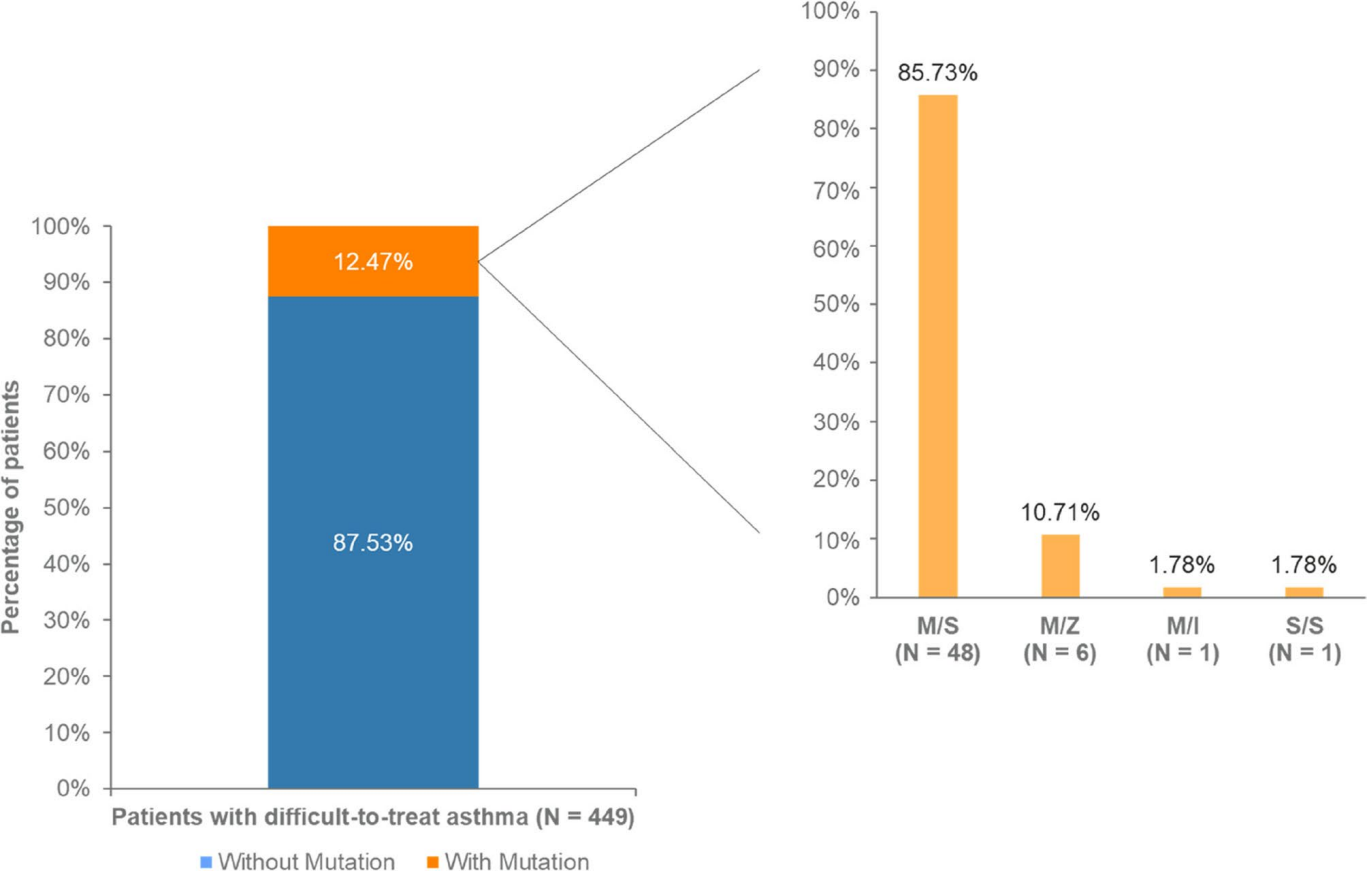
Prevalence of AAT gene mutation and its genotypes in patients with difficult-to-treat asthma in Colombia

Patients with difficult-to-treat asthma who met the eligibility criteria (*N* = 449) participated in the study; 73.1% were female with an average age of 56.1 ± 14.9 years (Table 1). It was found that 12.5% (*N* = 56) of the patients had some type of mutation in the gene coding for AAT (Fig. 1). Among these 56 patients, 85.7% (*N* = 48) had the M/S genotype, 10.7% (*N* = 6) had the M/Z genotype, 1.8% (*N* = 1) had the M/I genotype, and 1.8% (*N* = 1) had the S/S genotype (Fig. 1).

**Table 1:** General characteristics of the population

Characteristic	Total *N* = 449	Without Mutations *N* = 393 (87.5%)	With Mutations *N* = 56 (12.5%)	*P*-value
Age, years	56.1 ± 14.9	55.7 ± 14.8	55.5 ± 15.5	0.200
Sex				
Female	328 (73.1)	286 (72.8)	42 (75.0)	0.725
Male	121 (26.9)	107 (27.2)	14 (25.0)	
BMI, kg/m^2^	27.5 ± 4.9	27.6 ± 4.9	27.3 ± 4.6	0.726
Age of symptom onset	7.5 ± 14.5	7.5 ± 14.4	7.7 ± 15.7	0.810
Age at diagnosis	9.4 ± 17.0	9.6 ± 17.2	8.3 ± 15.9	0.403
Required emergency visit last year	79 (17.6)	72 (18.3)	7 (12.5)	0.285
Average emergency visits	2.1 ± 2.1	2.2 ± 2.2	1.6 ± 1.0	0.471
Severe exacerbations	130 (28.9)	116 (29.5)	14 (25.0)	0.486
Average severe exacerbations	2.2 ± 2.6	2.3 ± 2.8	1.4 ± 0.6	0.209
Required steroids during exacerbation	192 (42.8)	170 (43.3)	22 (39.3)	0.574
Average exacerbations requiring steroids	2.5 ± 2.9	2.5 ± 2.7	2.3 ± 4.0	0.725
Eosinophils cell/µL < 150 cell/µL ≥ 150 cell/µL	*n* = 321 300 (160–550) 73 (22.7) 248 (77.3)	*n* = 286 310 (170–560) 64 (25.7) 222 (77.6)	*n* = 35 280 (140–500) 9 (25.7) 26 (74.3)	0.317 0.657
IgE UI/ml ≤ 100 UI/ml > 100 UI/ml	*n* = 141 145 (46–464) 59 (41.8) 82 (58.2)	*n* = 126 158 (47–464) 52 (41.3) 74 (58.7)	*n* = 15 115 (32–717) 7 (46.7) 8 (53.3)	0.601 0.689
FeNO ppb ≤ 25 ppb > 25 ppb	*n* = 206 24 (13–42) 113 (54.8) 93 (45.2)	*n* = 181 24 (14–42) 96 (53.0) 85 (57.0)	*n* = 25 18 (9–55) 17 (68.9) 8 (32.0)	0.322 0.159

Values are presented as mean ± standard deviation, median (p_25_-p_75_), *N* (%)

When comparing the groups of patients with and without mutations, no significant differences were observed in the evaluated clinical characteristics, such as age, sex, body mass index, age of symptom onset, age at diagnosis, number of emergency visits, number of severe exacerbations, and inflammatory biomarkers, among others (Table 1).

Regarding comorbidities and environmental factors, there was no statistically significant difference in prevalence between patients with mutations and patients without mutations across the assessed variables (Table 2).

**Table 2:** Comorbidities and environmental factors

Variables	Total *N* = 449	Without Mutations *N* = 393	With Mutations *N* = 56	*P*-value	
Allergic rhinitis	232 (51.7)	204 (51.9)	28 (50.0)	0.789	
Sinusitis	116 (25.8)	102 (25.9)	14 (25.0)	0.879	
Atopic dermatitis	30 (6.7)	26 (6.6)	4 (7.1)	0.883	
Nasal polyps	81 (18.0)	71 (18.1)	10 (17.9)	0.970	
AERD	40 (8.9)	36 (9.2)	4 (7.1)	0.620	
GERD	138 (30.7)	124 (31.6)	14 (25.0)	0.320	
Vocal cord dysfunction	22 (4.9)	20 (5.1)	2 (3.6)	0.620	
History of smoking	85 (18.9)	74 (18.8)	11 (19.6)	0.884	
Pack-year index	11.6 ± 17.9 4.5 (0.8–11.5)	11.8 ± 18.7 4.5 (0.75–11.5)	9.9 ± 12.6 5 (1.8–19)	0.741	
Secondhand smoke	112 (24.9)	94 (23.9)	18 (32.1)	0.183	
Biomass exposure	60 (13.4)	54 (13.8)	6 (10.7)	0.529	
Occupational smoke exposure	59 (13.1)	55 (14.0)	4 (7.4)	0.156	

Values are presented as mean ± standard deviation, median (p25-p75), *N* (%)

*AERD* aspirin-exacerbated respiratory disease; *GERD* gastroesophageal reflux disease

In terms of lung function tests, data on the pre- and post-bronchodilator FEV1/FVC ratio, FEV1, and response to bronchodilator were recorded and no statistically significant differences were found between patients with and without mutations. No differences were documented in the scales for assessing asthma control [9–11]. In addition, dyspnea modified Medical Research Council (mMRC) score greater than or equal to 2 was more frequent in those who did not have the mutation, being a non-significant difference [12]. Additionally, those patients without mutation presented a higher percentage of early-onset asthma (41.2% vs. 32.1% *P* = 0.195), eosinophilic asthma (49.9% vs. 42.9% *P* = 0.327) and allergic asthma (63.7% vs. 55.4% *P* = 0.226), although these differences were not statistically significant (Table 3).

**Table 3:** Pulmonary function and prediction scales

Variables	Total *N* = 449	Without Mutations *N* = 393	With Mutations *N* = 56	*P*-value	
FEV1/FVC Pre B2	0.63 ± 0.11	0.63 ± 0.12	0.63 ± 0.10	0.804	
FEV1/FVC Post B2	0.66 ± 0.12	0.66 ± 0.12	0.65 ± 0.10	0.393	
BD Response (mL)	182.1 ± 175.4	184.9 ± 175.4	162.8 ± 175.9	0.378	
BD Response (%)	13.1 ± 16.1	13.1 ± 15.7	13.6 ± 18.7	0.805	
FEV1 < 80%	334 (74.6)	292 (74.5)	42 (75.0)	0.566	
**Prediction Scales**					
ACT Controlled Partially controlled Uncontrolled	174 (38.7) 158 (35.2) 117 (26.1)	150 (38.2) 139 (35.4) 104 (26.5)	24 (42.9) 19 (33.9) 13 (23.2)	0.777	
ACQ-5 Adequate control Partially controlled Inadequate control	*N* = 437 149 (34.1) 108 (24.7) 180 (41.2)	*N* = 382 124 (32.5) 99 (25.9) 159 (41.6)	*N* = 55 25 (45.5) 9 (16.4) 21 (38.2)	0.117	
TAI Good adherence Intermediate adherence Poor adherence	272 (63.3) 522 (12.1) 107 (24.8)	239 (63.2) 44 (11.6) 95 (25.1)	33 (62.3) 8 (15.1) 12 (22.6)	0.747	
mMRC 0–1 ≥ 2	314 (69.9) 135 (30.1)	272 (69.2) 121 (30.8)	42 (75.0) 14 (25.0)	0.377	
**Asthma Classification**					
Late-onset asthma	268 (59.7)	231 (58.8)	37 (66.1)	0.298	
Early-onset asthma	180 (40.1)	162 (41.2)	18 (32.1)	0.195	
Obesity-associated asthma	5 (1.1)	4 (1.0)	1 (1.8)	0.608	
AERD	11 (2.5)	11 (2.0)	0 (0.0)	0.305	
Non-classifiable asthma	6 (1.3)	4 (1.0)	2 (3.6)	0.119	
Eosinophilic asthma	214 (49.0)	190 (49.9)	24 (42.9)	0.327	
Allergic asthma	277 (62.7)	246 (63.7)	31 (55.4)	0.226	
Occupational smoke exposure	59 (13.1)	55 (14.0)	4 (7.4)	0.156	

Values are presented as mean ± standard deviation or *N*(%)

*ACT* Asthma Control Test; *ACQ* Asthma Control Questionnaire; *AERD* aspirin-exacerbated respiratory disease; *BD* Bronchodilator; *FEV1* Forced Expiratory Volume in the first second; *FVC* Forced Vital Capacity; *mMRC* Modified Medical Research Council; *TAI* Test of Adherence to Inhalers

Pharmacological treatment in patients with and without AAT gene mutations was found to be not statistically different, although patients without mutations had a higher percentage of long-acting muscarinic antagonist (LAMA) use than those with mutations (50.8% vs. 37.5%, *P* = 0.063). The percentage of patients vaccinated against influenza and pneumococcus, as well as those patients who participated in a pulmonary rehabilitation program, was similar in both groups (Table 4).

**Table 4:** Asthma treatment

Variables	Total *N* = 449	Without Mutations *N* = 393	With Mutations *N* = 56	*P*-value
GINA Step 3 Step 4 Step 5	49 (10.9) 202 (45.0) 198 (44.1)	42 (10.7) 179 (45.6) 172 (43.8)	7 (12.5) 23 (41.1) 26 (46.4)	0.801
Use of Oxygen	53 (11.8)	46 (11.7)	7 (12.5)	0.863
LABA	442 (98.4)	387 (98.5)	55 (98.2)	0.884
LAMA	220 (49.1)	199 (50.8)	21 (37.5)	0.063
Inhaled Steroid	385 (85.7)	337 (85.7)	48 (85.7)	0.994
SABA	366 (81.5)	317 (80.7)	49 (87.5)	0.217
SAMA	85(18.9)	76 (19.3)	9 (16.1)	0.559
Biological	150 (33.4)	130 (33.1)	20 (35.7)	0.696
Type of Biological (*N* = 150) Omalizumab Benralizumab Mepolizumab Dupilumab	*N* = 150 43 (28.7) 44 (29.3) 17 (11.3) 46 (30.7)	*N* = 130 36 (27.7) 39 (30.0) 14 (10.8) 41 (31.5)	*N* = 20 7 (35.0) 5 (25.0) 3 (15.0) 5 (25.0)	0.805
Influenza Vaccination (*N* = 446)	198 (44.4)	173 (44.4)	25 (44.6)	0.968
Pneumococcal Vaccination (*N* = 447)	114 (25.5)	99 (25.3)	15 (26.8)	0.814
Pulmonary Rehabilitation	163 (36.3)	142 (36.1)	21 (37.5)	0.842

Values are presented as *N* (%)

*GINA* Global Initiative for Asthma; *LABA* Long-Acting Beta Agonist; *LAMA* Long-Acting Muscarinic Antagonists; *SABA* Short-Acting Beta Agonist; *SAMA* Short-Acting Muscarinic Antagonist

## Discussion

This is the first study to report the prevalence and genotype of mutations of the AAT gene in patients with difficult-to-control asthma in Colombia. We found a prevalence of 12.5% for these mutations, which, when identified, have an impact on symptom control and patient mortality [16]. Our findings are similar to those reported by Eden et al., who found a prevalence of 10.5% in a diverse ethnic population with difficult-to-treat asthma in the United States [6]. Navasero et al., reported a prevalence of 10.0% in patients with moderate-to-severe asthma on or eligible for biologic therapy in a study conducted at two large private allergy practices in the United States [17]. In contrast, van Veen et al. reported a lower prevalence of 4.9% in a study conducted in the Netherlands, which included patients with severe asthma and different demographic characteristics compared to our study [8]. Additionally, Suarez et al. reported a prevalence of 22.4% in asthmatic patients allergic to house dust mites in Spain, who were younger and had less smoking history compared to our study population [18]. 

The main mutation genotype we found was M/S, which corresponds to 85.7%, followed by the M/Z genotype in 10.7%. Similar findings were reported in the study by Eden et al., who found the M/S genotype to be the most frequent in the Hispanic population at 75% [6]. The M/S genotype has been described as the most frequent in other respiratory diseases such as COPD. Data from a previous study in Colombia showed a prevalence of AATD in COPD of 13.0% and 8 genotypes in total [19]. 

Regarding the presence of mutations of the AAT gene in the entire study population (*N* = 449 patients), the M/S genotype was identified in 10.7% of the patients (48 out of 449). Considering that de Serres et al. estimated the prevalence of the M/S genotype to be 6.0% (2,671,230 out of 45,644,023 people) in the general Colombian population [20], our results suggest a possible enrichment of this AAT variant in the difficult-to-treat asthma population compared to the general Colombian population.

The presence of the *PiS* and *PiZ* variants of the *SERPINA1* gene has been associated with an increased risk of asthma exacerbations [21]. Studies have shown that individuals with the Pi*Z variant, in particular, have higher rates of emergency room visits, hospitalizations, and intensive care unit admissions due to asthma [22]. This highlights the importance of considering these genetic variants when evaluating patients with difficult-to-control asthma.

Our study did not find significant differences in clinical and paraclinical variables of patients with and without mutations, as published in previous studies [8]. Regarding demographic characteristics, the average age at asthma diagnosis was similar in the groups with and without mutations. However, patients without AATD mutations visited the emergency department more often in the past year, which was associated with a higher number of severe exacerbations and greater use of steroids.

Although no differences were observed in the asthma prediction and classification scales between the groups, a higher proportion of patients with mMRC dyspnea score greater than or equal to 2 was found in the non-mutated group, a finding that could also be related to the higher proportion of uncontrolled patients in the non-mutated group. Similar data has been previously reported [23]. This finding is in contrast with the data reported by Pini et al., where patients with an AAT mutation presented a lower asthma control according to the “Asthma Control Test” and “Asthma Control Questionnaire − 5” indices [3]. It is important to consider that in our population, no patients with severe AATD mutations were documented, which could have an impact on these findings.

Spirometric parameters were characteristic of patients with difficult-to-control asthma. More than 70% of the patients had an FEV1 < 80%, with no significant differences between the groups. These spirometric values coincide with those reported by Von Ehrenstein et al. [24] Additionally, although a trend towards increased use of LAMA for the treatment of asthma was observed among patients without mutations, comparable treatments were used in patients with and without mutations. Similar data have been previously described [25]. 

This is the first study that reports the prevalence and frequency of AAT mutation genotypes in patients with difficult-to-control asthma within a Colombian population, sampled from five respiratory disease centers. However, it is not without limitations. First, a limitation of our study is the lack of available serum levels of AAT. Second, in the description of the frequency of mutation genotypes, the Z/Z genotype was not found, probably due to the sample size. However, our objective was to determine the general prevalence of mutations and to describe the frequency of the genotypes found. Moreover, our findings are similar to previous studies in Spanish-speaking Latin Americans with similar sample sizes [18]. Third, we do not have imaging studies to describe the architecture of the lung parenchyma, but we were able to describe lung function, exacerbations, control scores, and level of treatment, which are variables with relevant clinical impact in patients with asthma. Fourth, although inflammatory biomarkers such as FeNO, total IgE, and blood eosinophil counts were included, a substantial proportion of patients did not have results available for these variables, limiting the scope of some analyses. Nevertheless, their inclusion even partially adds clinical value by providing insight into inflammatory phenotypes in this population. Finally, we excluded from our study patients with a previous AAT genotyping test. This exclusion criterion was intended to ensure that our study population consisted of individuals whose AAT status was unknown at the time of recruitment, thereby avoiding potential biases related to known AAT genotypes. However, this approach may have impacted the true prevalence of AATD. Therefore, this limitation should be considered when interpreting the study findings.

## Conclusions

This is the first study to identify the prevalence of AAT mutations in adult patients diagnosed with difficult-to-control asthma in Colombia, finding a prevalence of 12.5% and documenting four genotypes (M/S, M/Z, M/I, and S/S). Additional studies in other asthma classifications, as well as investigations that include AAT levels and diagnostic imaging, are required to better characterize this population and to identify potential treatable traits associated with these mutations.

## Data Availability

The data that support the findings of this study are available from the corresponding author upon reasonable request. The complete sequencing of the SERPINA1 gene is available in the GenBank Nucleotide Repository (accession number: NM_001127701.1). The genetic variation data used in the current study are available in the dbSNP repository, accession numbers: rs17580 (PI*S), rs28929474 (PI*Z), rs28931570 (PI*I), rs28931569 (PI*M procida), rs863225263 (PI*M malton), rs55819880 (PI*S iiyama), rs267606950 (PI*Q0 granite falls), rs751235320 (PI*Q0 west), rs199422211 (PI*Q0 bellingham), rs28929470 (PI*F), rs121912714 (PI*P Lowell), rs763023697 (PI*Q0 mattawa), rs764325655 (PI*Q0 clayton), and rs199422209 (PI*M heerlen).

## Abbreviations

AAT: Alpha-1 antitrypsin
AATD: alpha-1 antitrypsin deficiency
ACQ-5: Asthma Control Questionnaire − 5
ACT: Asthma Control Test
AERD: Aspirin Exacerbated Respiratory Disease
BD: Bronchodilator
CI: Confidence interval
COPD: Chronic obstructive pulmonary disease
FEV1: Forced Expiratory Volume in the first second
FVC: Forced Vital Capacity
GERD: Gastroesophageal Reflux Disease
GINA: Global Initiative for Asthma
ICS: inhaled corticosteroid
LABA: Long-acting B2 agonists
LAMA: Long-Acting Muscarinic Antagonists
TAI: Inhaler Adherence Test
SABA: Short-acting beta agonist
SAMA: Short-acting muscarinic antagonist
